# *Arabidopsis* NAC Transcription Factor JUNGBRUNNEN1 Exerts Conserved Control Over Gibberellin and Brassinosteroid Metabolism and Signaling Genes in Tomato

**DOI:** 10.3389/fpls.2017.00214

**Published:** 2017-03-07

**Authors:** Sara Shahnejat-Bushehri, Annapurna D. Allu, Nikolay Mehterov, Venkatesh P. Thirumalaikumar, Saleh Alseekh, Alisdair R. Fernie, Bernd Mueller-Roeber, Salma Balazadeh

**Affiliations:** ^1^Institute of Biochemistry and Biology, University of Potsdam,Potsdam, Germany; ^2^Max Planck Institute of Molecular Plant Physiology,Potsdam, Germany

**Keywords:** *Arabidopsis*, tomato, fruit, growth, transcription factor, gibberellic acid, brassinosteroid, DELLA proteins

## Abstract

The *Arabidopsis thaliana* NAC transcription factor JUNGBRUNNEN1 (AtJUB1) regulates growth by directly repressing *GA3ox1* and *DWF4*, two key genes involved in gibberellin (GA) and brassinosteroid (BR) biosynthesis, respectively, leading to GA and BR deficiency phenotypes. AtJUB1 also reduces the expression of *PIF4*, a bHLH transcription factor that positively controls cell elongation, while it stimulates the expression of *DELLA* genes, which are important repressors of growth. Here, we extend our previous findings by demonstrating that *AtJUB1* induces similar GA and BR deficiency phenotypes and changes in gene expression when overexpressed in tomato (*Solanum lycopersicum*). Importantly, and in accordance with the growth phenotypes observed, AtJUB1 inhibits the expression of growth-supporting genes, namely the tomato orthologs of *GA3ox1*, *DWF4* and *PIF4*, but activates the expression of *DELLA* orthologs, by directly binding to their promoters. Overexpression of *AtJUB1* in tomato delays fruit ripening, which is accompanied by reduced expression of several ripening-related genes, and leads to an increase in the levels of various amino acids (mostly proline, β-alanine, and phenylalanine), γ-aminobutyric acid (GABA), and major organic acids including glutamic acid and aspartic acid. The fact that AtJUB1 exerts an inhibitory effect on the GA/BR biosynthesis and *PIF4* genes but acts as a direct activator of *DELLA* genes in both, *Arabidopsis* and tomato, strongly supports the model that the molecular constituents of the JUNGBRUNNEN1 growth control module are considerably conserved across species.

## Introduction

Gibberellins (GAs) and brassinosteroids (BRs) play important roles in the regulation of plant growth and development. Because of their biological relevance, many key aspects of the metabolism and signaling of both phytohormones have been revealed in the past (Depuydt and Hardtke, 2011; Bai et al., 2012). GAs control processes like for example seed germination, leaf expansion, shoot elongation, flowering, fruit set and growth (Olszewski et al., 2002; Yamaguchi, 2008; Mutasa-Göttgens and Hedden, 2009; Seymour et al., 2013). Similarly, BRs function as growth-promoting hormones mediating many aspects of plant growth and development including the stimulation of growth through cell division and cell elongation, and the adaptation to environmental cues (Clouse, 2011). Mutants deficient in BR biosynthesis or perception exhibit various degrees of phenotypic similarity to GA-deficient mutants including dwarfism, short petioles and hypocotyls, curled dark-green leaves, and delayed flowering (Clouse and Sasse, 1998; Fleet and Sun, 2005).

With respect to the later steps of GA biosynthesis, GA 20-oxidase (GA20ox) and GA 3-oxidase (GA3ox) are particularly important for controlling the levels of bioactive GAs (Phillips et al., 1995; Xu et al., 1995; Yamaguchi et al., 1998). The bioactive GAs produced by GA3ox enzymes are perceived by a soluble GA receptor, GIBBERELLIN-INSENSITIVE DWARF1 (GID1) (Ueguchi-Tanaka et al., 2005). The binding of GA to GID1 triggers the destruction of DELLA proteins by the ubiquitin-proteasome pathway, or their inactivation through a degradation-independent mechanism (Ariizumi et al., 2008, 2013; Ueguchi-Tanaka et al., 2008). DELLAs are major transcriptional growth repressors (Martí et al., 2007; Bai et al., 2012). In the absence of active GAs, DELLAs accumulate allowing them to interact with the transcription factors (TFs) PHYTOCHROME-INTERACTING FACTOR4 (PIF4) and BRASSINAZOLE-RESISTANT1 (BZR1) to inhibit their DNA-binding ability; this restricts cell elongation controlled by the two TFs thereby resulting in reduced plant growth (De Lucas et al., 2008; Bai et al., 2012).

Gibberellins and DELLAs have also been reported to control reproductive organ size and fruit development in several species (Martí et al., 2007; Dorcey et al., 2009; Fuentes et al., 2012). In tomato (*Solanum lycopersicum*), overexpression of *SlDELLA* (which is identical to *SlGAI*) suppresses fruit development by inhibiting cell expansion (Martí et al., 2007).

Brassinosteroids are synthesized from campesterol via two pathways, the early and the late C-6 oxidation pathways (Fujioka and Sakurai, 1997). DWARF4 (DWF4) encodes a cytochrome P450 protein that catalyzes sterol C-22 α-hydroxylation, a rate-limiting step in BR biosynthesis (Choe et al., 1998, 2001; Kim et al., 2006; Divi and Krishna, 2010). The BR signal is perceived by a membrane-bound receptor kinase, BRASSINOSTEROID INSENSITIVE1 (BRI1). BRs bind to BRI1 and initiate a signal transduction pathway which ultimately results in the dephosphorylation and thereby stabilization of BZR1 and BRI1-EMS SUPPRESSOR1 (BES1) (Wang et al., 2002; He et al., 2005; Yin et al., 2005), two TFs regulating BR-responsive genes to promote growth (Sun et al., 2010; Clouse, 2011; Yu et al., 2011). BR and GA signals are integrated through the direct interaction of BZR1 and BES1 with DELLAs, and they enhance each other’s signaling through inactivation and degradation of DELLA proteins, respectively (Bai et al., 2012; Gallego-Bartolomé et al., 2012; Li et al., 2012). In the presence of BRs, active forms of BZR1 and BES1 attenuate the transcriptional activity of DELLAs. On the other hand, in the absence of GA, accumulation of DELLAs and their binding to BZR1 or BES1 prevents their dephosphorylation and thus results in their inactivation. BR-deficient and -insensitive mutants have, e.g., been identified in *Arabidopsis thaliana*, pea (*Pisum sativum*), tomato (*Lycopersicum solanum*), and rice (*Oryza sativa*) (Clouse and Sasse, 1998; Bishop, 2003; Vert et al., 2005; Clouse, 2011). BR biosynthesis pathways appear to be conserved between *Arabidopsis*, tomato, and pea (Bancoş et al., 2002). Molecular genetic manipulation of BR biosynthesis and signaling has been used to improve crop yield and stress tolerance (Divi and Krishna, 2009). It has been shown that BR mutants in rice and barley have increased grain yield and productivity due to erect leaves and reduced plant height (Chono et al., 2003; Morinaka et al., 2006). Controlling plant architecture and height to obtain miniature ornamental plants has been achieved by co-suppression of genes controlling GA and BR biosynthesis pathways (Xie et al., 2014). Several studies have shown that BRs are required for normal fruit development. For example, BR levels are high in developing fruits of tomato (Montoya et al., 2005). Furthermore, tomato fruit ripening is accelerated by application of BRs, but is delayed by treatment with the BR biosynthesis inhibitor brassinazole (Vidya Vardhini and Rao, 2002; Lisso et al., 2006). In tomato, the BR-deficient *dwarf* mutant shows delayed flowering and fruit ripening. Dry mass content, starch and sugar levels are reduced in *dwarf* tomato fruits, but are rescued by BR application, indicating that BRs are required for normal fruit development in this species (Lisso et al., 2006).

We recently identified the *Arabidopsis thaliana* NAC transcription factor JUNGBRUNNEN1 (JUB1; in the following called AtJUB1) as a further element of the GA/BR control network. More specifically, AtJUB1 suppresses the expression of *GA3ox1* and *DWF4*, two central genes of GA and BR biosynthesis, respectively, by directly binding to their promoters. Furthermore, AtJUB1 directly activates the expression of the DELLA encoding genes *GAI* and *RGL1*. The repression of the phytohormone biosynthesis genes and the activation of the *DELLA* genes leads to an accumulation of DELLA proteins in *AtJUB1* overexpressors and growth characteristics typical of low-GA and low-BR mutants, mediated by the inhibition of cell elongation (Shahnejat-Bushehri et al., 2016). AtJUB1 further inhibits cell elongation by directly suppressing the expression of *PIF4* (Shahnejat-Bushehri et al., 2016).

Here, we report that overexpression of *AtJUB1* in tomato results in GA and BR deficiency phenotypes similar to those observed in *Arabidopsis AtJUB1* overexpressors. Furthermore, we show that AtJUB1 directly regulates the expression of tomato genes orthologous to *GA3ox1*, *DWF4*, *GAI*, and *PIF4* from *Arabidopsis*. Our study thus reveals considerable similarity of the GA/BR-related gene regulatory network controlled by JUB1 across species.

## Materials and Methods

### General

Tomato orthologs of *Arabidopsis* genes were identified using the PLAZA 3.0 database^1^ (Proost et al., 2015). Gene annotations were done using the PLAZA 3.0 and Sol Genomics^2^ database, and information taken from the literature. Quantitative real-time polymerase chain reaction (qRT-PCR) primers were designed using QuantPrime (Arvidsson et al., 2008); only primer pairs giving high PCR amplification efficiencies tested on at least 20 different RNAs isolated from diverse tomato tissues were chosen for the experiments performed here. Sequences of primers used for qRT-PCR and chromatin immunoprecipitation-polymerase chain reaction (ChIP-PCR) are given in **Supplementary Table S1**.

### Plant Material and Growth Conditions

To generate *AtJUB1-OX* tomato plants, *S. lycopersicum* cv. Moneymaker was transformed with the *35S:AtJUB1-GFP* construct previously reported (Wu et al., 2012) by *Agrobacterium*-mediated transformation. Seeds were germinated on full-strength Murashige-Skoog (MS) medium containing 2% (w/v) sucrose in a plant growth cabinet at 22°C, at a 16/8 h light (100–120 mmol photons m^−2^ s^−1^)/dark cycle. Two-week-old seedlings were transplanted to soil (potting and quartz sand; 2:1 [v/v]) and grown in the greenhouse at 25°C under a 16/8 h light (500 mmol photons m^−2^ s^−1^)/dark cycle, in individual pots (18 cm diameter). After transfer to soil, plants were fertilized with 5 g/pot Complesal Perfekt (15% N, 5% P_2_O_5_, 20% K_2_O, 2% MgO, 7% S; Manna, Duesseldorf, Germany); two further fertilizations (with each 4 g/pot) followed when the first flowers opened, and when the first stage B fruits appeared.

### Quantitative Real-Time PCR

Total RNA from frozen powdered tissue (200 mg fruit, or 100 mg leaf) was isolated using Trizol reagent (Life Technologies). cDNA synthesis and qRT-PCR were performed as described (Caldana et al., 2007; Balazadeh et al., 2008). Prior to cDNA synthesis, RNA was treated with DNase (Ambion) to eliminate genomic DNA contamination. Purity of DNase-treated RNA was assessed with intron-specific primers on gene *Solyc01g056340* using qRT-PCR. Prior to gene expression analysis, cDNA quality was determined using GAPDH-3′ and GAPDH-5′ (*Solyc04g009030*) primer pairs. Real-time qPCR was performed with SYBR Green (Applied Biosystems) and gene-specific primers, using an ABI PRISM 7900HT sequence detection system (Applied Biosystems). *GAPDH* served as reference gene for data analysis.

### Chromatin Immunoprecipitation (ChIP)

The chromatin extract was isolated as previously described (Kaufmann et al., 2010) from leaves of mature *AtJUB1-OX* (*35S:AtJUB1-GFP*) plants, and wild type plants served as control. Anti-GFP antibody (Abcam) was used to immunoprecipitate protein–DNA complexes. After protein digestion, DNA was purified by QIAquick PCR Purification kit (Qiagen) and analyzed by qPCR using primers flanking AtJUB1 binding sites in tomato promoters. As a negative control for ChIP-qPCR data analysis, we used a primer pair amplifying the 5′ upstream regulatory region of *Solyc01g090460*, which lacks an AtJUB1 binding site. ChIP-qPCR data were analyzed as described (Wu et al., 2012).

### Microarray Analysis

Total RNA was isolated from *AtJUB1-OX* and wild type fruits at MG and B+7 stages using Trizol reagent and quality-checked as for qRT-PCR experiments. Three μg of quality-checked RNA was processed for transcriptome profiling using GeneChip Tomato Genome Arrays (Affymetrix). Probe preparation and microarray hybridizations were performed by ATLAS Biolabs (Berlin, Germany). Data analysis was performed as in Balazadeh et al. (2010). Expression of differentially expressed genes (transgenic versus wild type) was reassessed by qRT-PCR using RNA isolated from plants of two independent biological experiments. Microarray expression data are available from the NCBI Gene Expression Omnibus (GEO) repository^3^ under accession number GSE87507.

### Profiling of Primary Metabolites by GC–MS

Fruit materials were extracted in 100% methanol at 70°C for 15 min. After centrifugation, the resultant supernatant was dried under vacuum, and the residue was derivatized for 120 min at 37°C (in 50 μl of 20 mg ml^-1^ methoxyamine hydrochloride in pyridine) followed by a 30 min treatment at 37°C with 50 μl of *N*-methyl-*N*-(trimethylsilyl)-trifluoroacetamide (MSTFA). The gas chromatography–mass spectrometry (GC–MS) system used was a gas chromatograph coupled to a time-of-flight mass spectrometer (Pegasus III, Leco). An autosampler system (PAL) injected the samples. Helium was used as carrier gas at a constant flow rate of 2 ml s^−1^ and gas chromatography was performed on a 30 m DB-35 column. The injection temperature was 230°C and the transfer line and ion source were set to 250°C. The initial temperature of the oven (85°C) increased at a rate of 15°C min^−1^ up to a final temperature of 360°C. After a solvent delay of 180 s mass spectra were recorded at 20 scans s^−1^ with m/z 70–600 scanning range. Chromatograms and mass spectra were evaluated by using Chroma TOF 1.0 (Leco) and TagFinder 4.0 software (Roessner et al., 2001; Schauer et al., 2005).

## Results

### Tomato Plants Overexpressing *AtJUB1* Exhibit Morphological Characteristics of GA- and BR-Deficient Mutants

**FIGURE 1:**
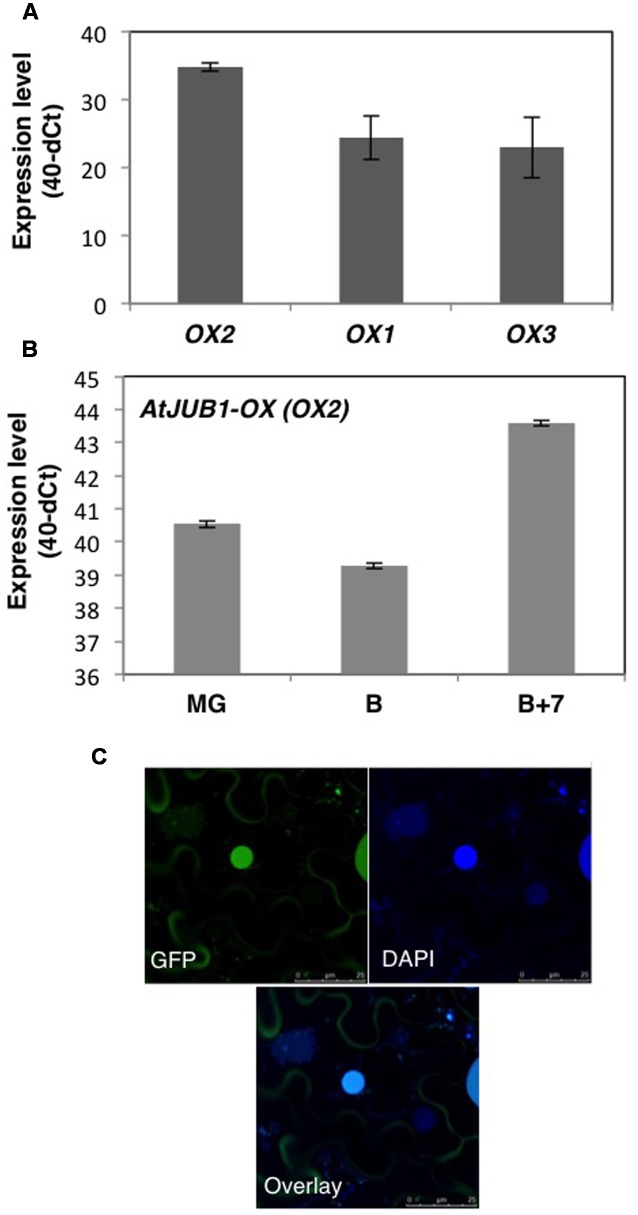
**Analysis of *AtJUB1-OX* transgenic plants. (A)** Expression level of *AtJUB1* in leaves of transgenic lines OX1, OX2, and OX3 compared to wild type (WT). Mean ± SD (*n* = 3). **(B)** Expression of *AtJUB1* in fruits of line OX2 at mature green (MG), breaker (B), and breaker+7d (B+7) stages. Mean ± SD (*n* = 3). For quantitative real-time polymerase chain reaction (qRT-PCR) analysis, values are expressed as the difference between an arbitrary value of 40 and dCt, so that high 40-dCt value indicates high gene expression level. **(C)** Nuclear localization of GFP-tagged AtJUB1 protein in leaves of transgenic tomato plants, imaged by confocal microscopy. Top left, fluorescence microscopy; top right, DAPI staining; bottom, overlay. Some AtJUB1-GFP protein appears to reside in the cytoplasm surrounding the central vacuole, likely due to incomplete nuclear import.

**FIGURE 2:**
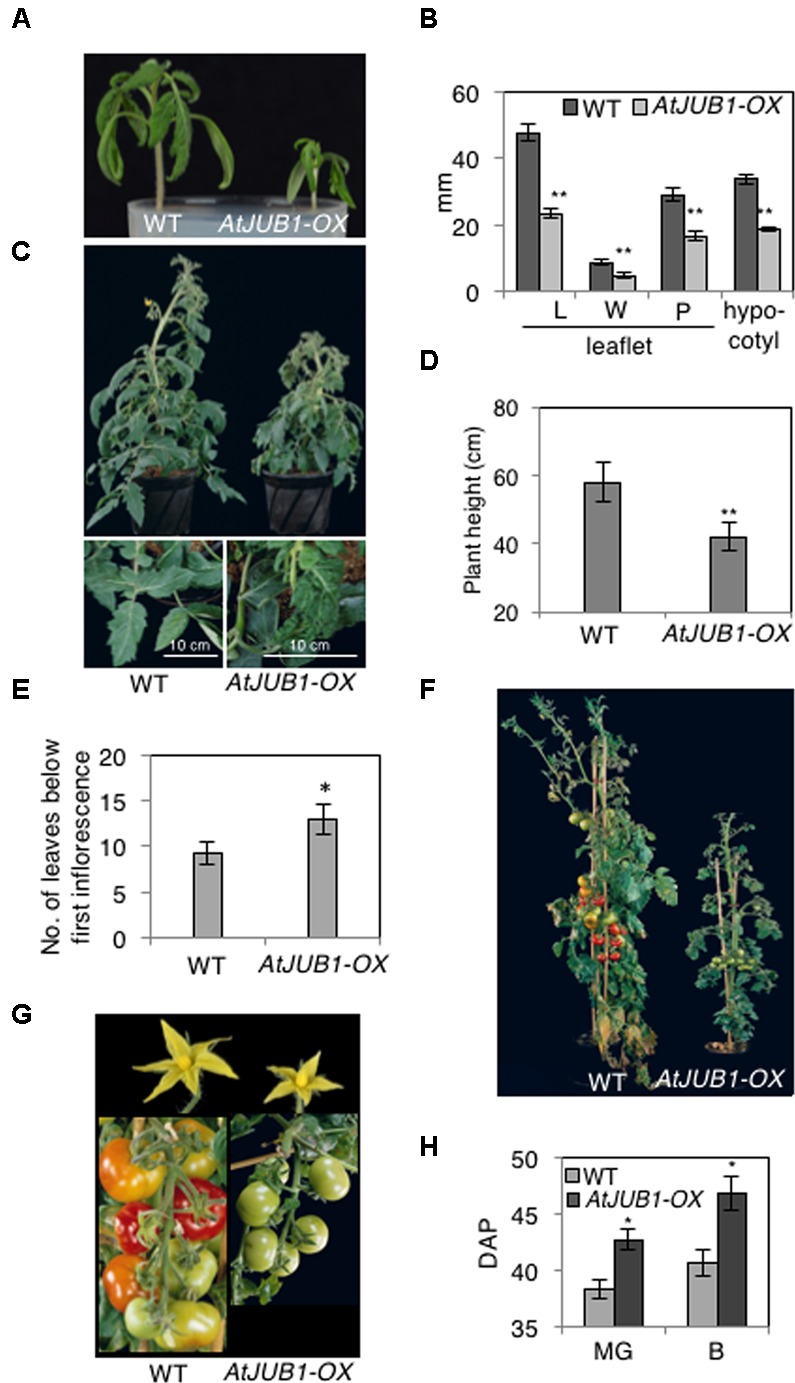
**Phenotype of *AtJUB1-OX* tomato plants at different developmental stages. (A)** Reduced growth in 3-week-old *AtJUB1-OX* transgenic plants strongly overexpressing *AtJUB1*, compared to wild type (WT). **(B)** Leaf parameters: length (L) and width (W) of leaflet blade; petiolule (P) length; hypocotyl length of 3-week-old *AtJUB1-OX* and WT tomato seedlings. Leaf parameters were determined for the two subterminal leaflets of leaf number 1. **(C)** Vegetative growth phenotype of 6-week-old plants and their leaves (lower panel). **(D)** Height of plants. **(E)** Flowering time of *AtJUB1-OX* and WT plants measured by counting the number of leaves on the date that the first inflorescence arose. **(F)** Phenotype of plants at reproductive stage. **(G)** Flowers and fruits of plants shown in **(F)**. **(H)** Fruit ripening was scored in *AtJUB1-OX* plants compared to WT by counting the days after pollination (DAP) to reach the mature green and breaker stages. Data in graphs represent mean ± SD (*n* = 10) and ^∗^*p* < 0.05, ^∗∗^*p* < 0.01 by Student’s *t*-test. Asterisks denote significant differences to WT.

To understand the potential role of the JUNGBRUNNEN1 TF in tomato, we generated transgenic plants (*S. lycopersicum* L. cv. Moneymaker) overexpressing *AtJUB1-GFP* (Wu et al., 2012) under the control of the Cauliflower Mosaic Virus (CaMV) *35S* promoter. We previously showed that the JUB1-GFP fusion protein is fully functional when expressed in plants (Wu et al., 2012). We obtained more than 20 independent transgenic lines and determined the level of *AtJUB1* expression in some of them by qRT-PCR in leaves (**Figure 1A**; lines OX1, OX2, and OX3) and fruits of different developmental stages (**Figure 1B**; OX2; thereafter called *AtJUB1-OX*). The formation of full-length *AtJUB1* transcript in transgenic tomato plants was confirmed by PCR analysis of cDNA of leaves and fruits, using primers specific for *AtJUB1* (**Supplementary Figures S1A,B**). Accumulation of AtJUB1-GFP fusion protein in nuclei of *AtJUB1-OX* tomato leaves was confirmed by confocal microscopy (**Figure 1C**). *AtJUB1-OX* lines displayed distinct morphological changes compared to the Moneymaker wild type (**Figure 2**). Strong *AtJUB1* overexpressors (line OX2) had smaller shoots than wild type and developed smaller leaves (**Figures 2A,B**), while growth was not much reduced in plants moderately overexpressing *AtJUB1*, including OX1 and OX3 (not shown). Furthermore, *AtJUB1-OX* plants developed curly and dark-green leaves, a phenotype not observed in the wild type (**Figure 2C**). Additionally, overexpression of *AtJUB1* in tomato delayed flowering, similar to *Arabidopsis* (Shahnejat-Bushehri et al., 2016). *AtJUB1-OX* tomato plants flowered when the main shoot had ∼13 leaves beneath the first inflorescence compared to ∼9 leaves in the wild type (**Figure 2E**). At the productive stage, *AtJUB1-OX* plants had smaller flowers and fruits than the wild type (**Figures 2F,G**), and fruit development and ripening were delayed. Fruits of *AtJUB1-OX* plants reached the mature green stage (MG) 4 days later than wild type tomatoes (measured as days after pollination) and ripening of *AtJUB1-OX* fruits was delayed by ∼6 days (**Figure 2H**). Upon visual inspection, *AtJUB1-OX* fruits showed reduced pigmentation at the breaker+7d (B+7) stage compared to wild type fruits at the same stage (not shown). Taken together, many of the growth phenotypes observed in *AtJUB1-OX* tomato plants were similar to those of *Arabidopsis* plants overexpressing *AtJUB1*, suggesting that JUB1 controls a similar set of growth-related genes in both species possibly including genes affecting hormone metabolism and signaling (Shahnejat-Bushehri et al., 2016).

### AtJUB1 Directly Regulates GA- and BR-Related Genes in Tomato

**FIGURE 3:**
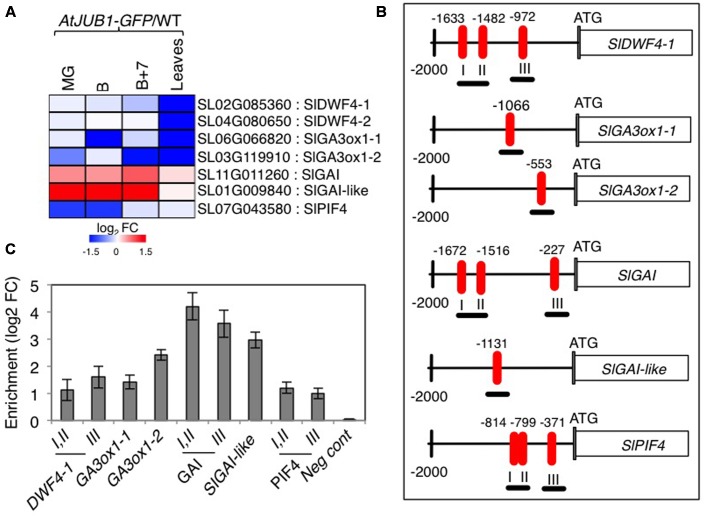
**Direct regulation of GA- and BR-associated genes by AtJUB1. (A)** Heat map showing the expression of orthologs of *Arabidopsis DWF4*, *GA3ox1*, *GAI*, *RGL1*, and *PIF4* in tomato line OX2 at mature green (MG), breaker (B), breaker+7d (B+7) stages and leaves of mature plants compared to wild type (WT). The log_2_ fold change scale is indicated under the heat map. Complete data are given in **Supplementary Table S2**. **(B)** Schematic diagram showing positions of AtJUB1 binding sites (numbered I, II and III if three sites were present) in the promoters of genes orthologous to the AtJUB1 targets in *Arabidopsis*. ChIP amplicons are indicated as black thick underlines. **(C)** ChIP assays showing the direct binding of AtJUB1 to the promoters of *SlDWF4-1*, *SlGA3ox1-1*, *SlGA3ox1-2*, *SlGAI*, *SlGAI-like* and *SlPIF4*. Values were normalized to the values for *Solyc01g090460* (promoter lacking AtJUB1 binding site), as a negative control. Error bars represent mean ± SD (two independent biological replicates, each with three technical replicates). FC, fold change.

Previously, we reported that AtJUB1 extends longevity, dampens intracellular H_2_O_2_ level, and enhances tolerance to various abiotic stresses partly through direct transcriptional regulation of *DREB2A*, an important TF involved in the response to various abiotic stresses (Sakuma et al., 2006a,b; Wu et al., 2012). Furthermore, AtJUB1 restricts growth and primes plants for stress tolerance in a DELLA-dependent manner by regulating a complex transcriptional module composed of key components of GA and BR pathways. In *Arabidopsis*, AtJUB1 directly suppresses the expression of *GA3ox1* (GA biosynthesis), *DWF4* (BR biosynthesis), and *PIF4* (regulation of cell elongation), but activates the expression of the *GAI* and *RGA* DELLA genes (Shahnejat-Bushehri et al., 2016). Considering the growth phenotypes observed for the *AtJUB1-OX* tomato plants (see above), we tested whether similar genes are targets of AtJUB1 in tomato. First, we checked the expression of tomato orthologs of *Arabidopsis DWF4*, *GA3ox1*, *GAI* and *RGL1* in *AtJUB1-OX* tomato leaves and in fruits at the MG, B and B+7 stages. As shown in **Figure 3A**, expression of the *AtDWF4* tomato orthologs *Solyc02g085360* (denoted *SlDWF4-1* in the following) and *Solyc04g080650* (*SlDWF4-2*) was not altered in fruits of *AtJUB1-OX* fruits at all stages tested, but was strongly reduced in leaves of mature plants compared to wild type. Similarly, expression of the *AtGA3ox1* tomato orthologs *Solyc06g066820* (denoted *SlGA3ox1-1* in the following) and *Solyc03g119910* (*SlGA3ox1-2*) was significantly downregulated in leaves of mature *AtJUB1-OX* plants. Moreover, *SlGA3ox1-1* was lower expressed in fruits of *AtJUB1-OX* plants at the breaker (B) stage, while expression of *SlGA3ox1-2* was decreased in MG and B+7 fruits compared to wild type fruits of the same stage. Interestingly, expression of the tomato DELLA genes *Solyc11g011260* (*SlGAI*) and *Solyc01g009840* (*SlGAI-like*) was slightly and strongly upregulated, respectively, in MG, B and B+7 fruits of *AtJUB1-OX* plants, consistent with the reduced size of the fruits. Expression of *SlGAI* and *SlGAI-like* was not altered in leaves of *AtJUB1-OX* plants relative to wild type. Expression of *Solyc07g043580* (*SlPIF4*), the positive regulator of cell elongation, was strongly suppressed by AtJUB1 in MG and B, but not affected in B+7 fruits or leaves of *AtJUB1-OX* plants.

The fact that JUB1 regulates orthologous genes in *Arabidopsis* and tomato when overexpressed indicates that this TF obeys a conserved function in plants as part of a complex regulatory network associated with GA and BR pathways. To consolidate this conclusion we next analyzed the promoters of selected GA- and BR-related genes whose expression is affected by *AtJUB1* overexpression (shown in **Figure 3A** and **Supplementary Table S2**), and found that *SlDWF4-1*, *SlGA3ox1-1*, *SlGA3ox1-2*, *SlGAI* and *SlGAI-like* harbor the conserved AtJUB1 binding site (Wu et al., 2012) in their 5′ upstream regulatory regions (**Figure 3B**). An AtJUB1 binding site is also present in the promoter of *SlPIF4* (**Figure 3B**). To test whether AtJUB1 is able to bind to the selected putative target genes in tomato, we performed chromatin immunoprecipitation followed by qPCR using leaves of mature *AtJUB1-OX* plants. Binding of AtJUB1 was observed for all promoters tested, while no binding was detected for a promoter region lacking an AtJUB1 binding site (negative control) (**Figure 3C**). Taken together, our data indicate a direct transcriptional regulation of *SlDWF4-1*, *SlGA3ox1-1*, *SlGA3ox1-2*, *SlGAI*, *SlGAI-like* and *SlPIF4* by AtJUB1 *in vivo*.

### Differentially Expressed Genes in *AtJUB1* Overexpressing Tomato Fruits

**FIGURE 4:**
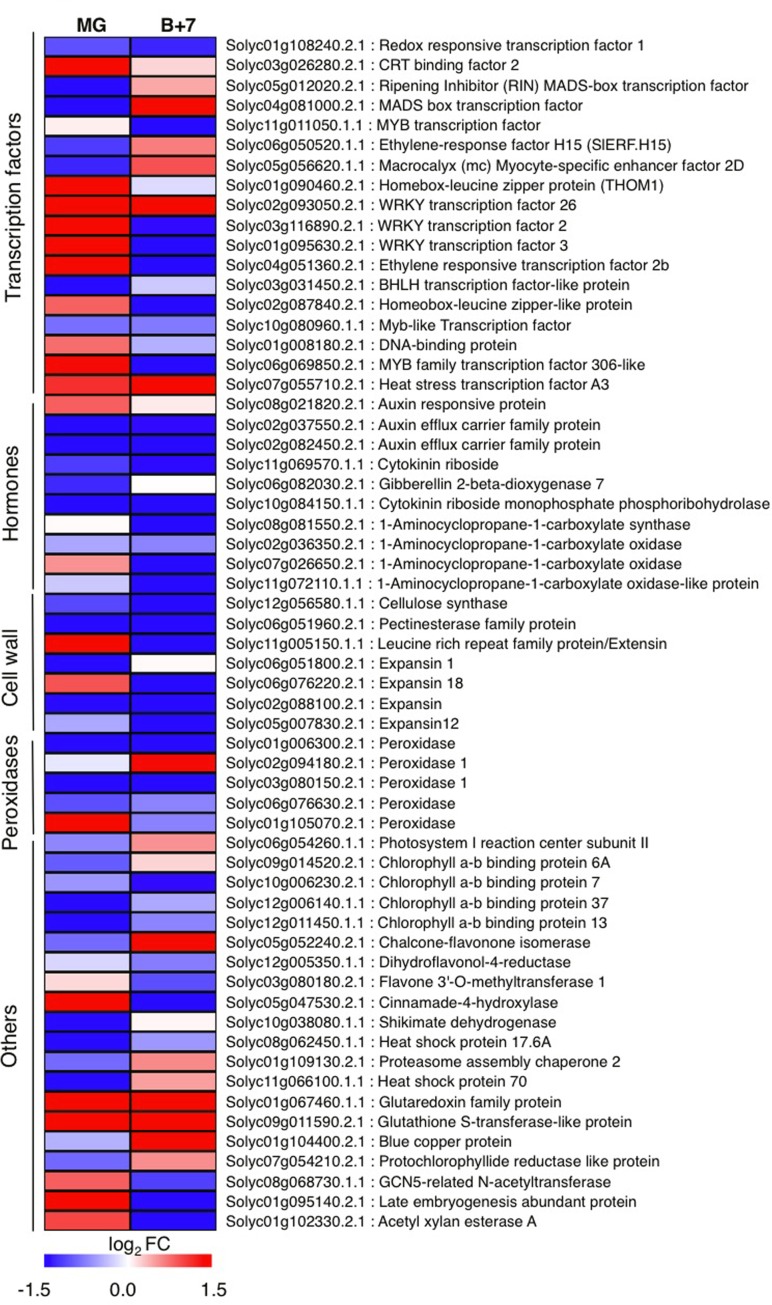
**Quantitative real-time polymerase chain reaction analysis of AtJUB1-regulated genes in tomato fruits.** Heat map showing differentially expressed genes in *AtJUB1-OX* fruits compared to wild type. Means of three experiments are shown; complete data are given in **Supplementary Table S3**. The log_2_ fold change scale is indicated under the heat map. Transcription factors, hormone metabolism-related, cell wall-related and peroxidase categories are shown.

To identify genes globally affected by AtJUB1 during fruit development and ripening, we compared the transcriptomes of *AtJUB1-OX* and wild type fruits at MG and B+7 stages, using microarray analysis. We selected 92 genes that were differentially expressed in MG and/or B+7 fruits of *AtJUB1-OX* compared to wild type (at a 1.5-fold cut-off in increase or decrease of gene expression) and re-analyzed their expression in additional biological replicates by qRT-PCR, which confirmed altered expression of 60 genes (**Figure 4** and **Supplementary Table S3**). The AtJUB1-regulated genes fall into different functional categories including TFs, cell wall-modifying enzymes, antioxidant enzymes, enzymes involved in flavonoid biosynthesis, and hormone-related genes. Among the TFs, *RIPENING INHIBITOR* (*RIN*; *Solyc05g012020*), a MADS-box TF with a key function in fruit ripening, was significantly downregulated in *AtJUB1-OX* tomato fruits at MG stage. Similarly, *ETHYLENE RESPONSE FACTOR H15* (*SlERF.H15*; *Solyc06g050520*), which shows increased expression at the onset of ripening and together with other *ERFs* was suggested to be involved in regulating the ripening process (Liu et al., 2016), was downregulated in *AtJUB1-OX* fruits at MG stage. Furthermore, expression of cell wall-related genes including a pectin esterase family protein (*Solyc06g051960*), a cellulase synthase (*Solyc12g056580*), and expansins (*Solyc06g051800*, *Solyc02g088100*, and *Solyc05g007830*) was reduced in *AtJUB1-OX* fruits. A group of genes associated with cellular oxidation and peroxidation processes, including several peroxidases (*Solyc01g006300*, *Solyc03g080150*, *Solyc06g076630*), were lower expressed in *AtJUB1-OX* fruits. Peroxidases are considered to affect fruit softening and ripening (Kumar et al., 2016). Expression of *Solyc12g005350*, encoding dihydroflavonol-4-reductase, a key gene in anthocyanin biosynthesis (Holton and Cornish, 1995), is slightly decreased in *AtJUB1-OX*. Among hormone-related genes, expression of 1-aminocyclopropane-1-carboxylate (ACC) synthase (*ACS*; *Solyc08g081550*) and ACC oxidases (*ACO*; *Solyc02g036350*, *Solyc07g026650*, *Solyc11g072110*) is either not altered or repressed in *AtJUB1-OX* fruits. These genes are known to be involved in ethylene biosynthesis and their expression is induced during fruit ripening (Barry et al., 2000). Moreover, *Solyc02g037550* and *Solyc02g082450*, members of the auxin efflux carrier family, are significantly downregulated in *AtJUB1-OX* fruits at stages MG and B+7. These proteins are involved in the distribution of auxin, a growth hormone controlling many aspects of fruit development and ripening (Gillaspy et al., 1993; Srivastava and Handa, 2005). Next, we searched for the binding site of *AtJUB1* in the promoters of the differentially expressed genes. The absence of the full AtJUB1 binding site in the 5′ upstream regulatory regions of the 60 genes differentially expressed between *AtJUB1-OX* and wild type suggests that AtJUB1 indirectly, e.g., in conjunction with hormonal networks, regulates their expression.

### AtJUB1 Affects Metabolite Profiles in Tomato Fruits

**FIGURE 5:**
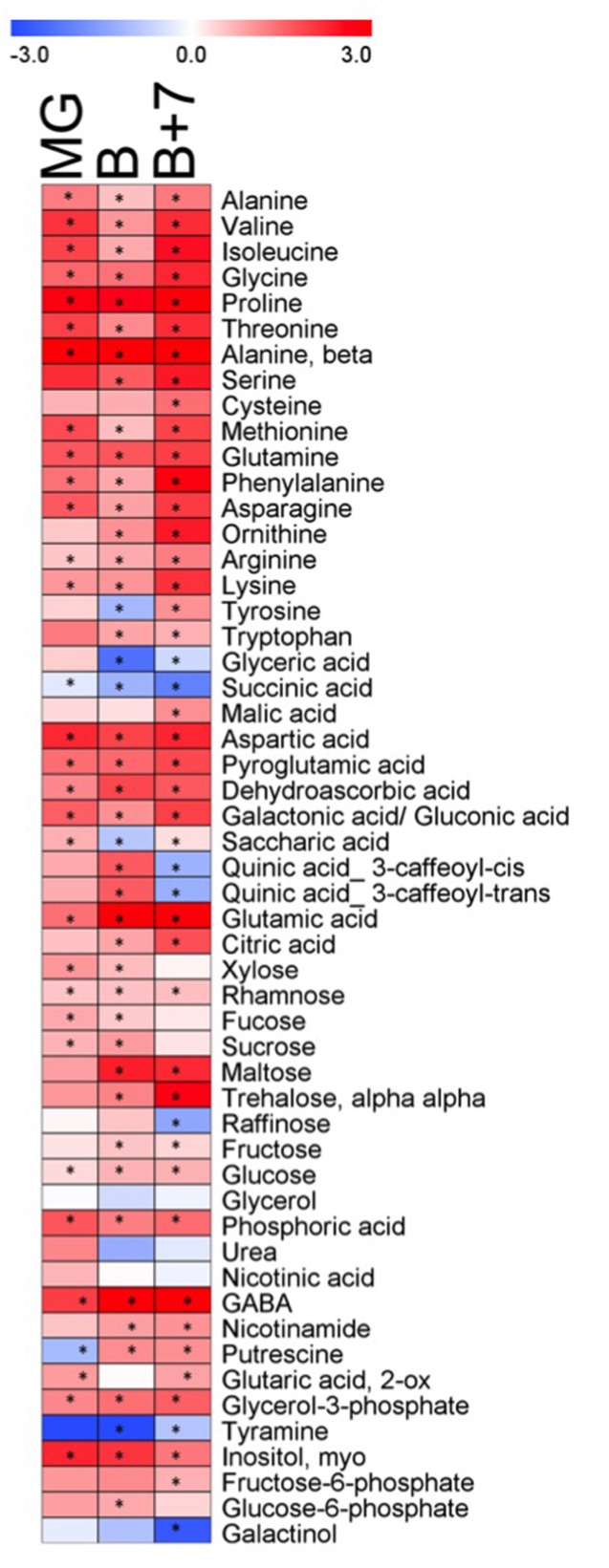
**Heat map showing the metabolite changes in fruits of *AtJUB1-OX* plants in comparison with wild type at three ripening stages.** Mature green fruit (MG), breaker stage (B), and red ripe fruit (B+7) were analyzed. Red or blue indicate that the metabolite content is increased or decreased, respectively. Asterisks denote significant difference (*p* ≤ 0.01, Student’s *t*-test) in *AtJUB1-OX* compared to wild type; complete data are given in **Supplementary Table S4**.

Metabolite profiling was performed on fruits of *AtJUB1-OX* and wild type plants at three independent ripening stages: mature green (MG), breaker (B) and red ripe fruit (B+7). Metabolites were separated and quantified by GC–MS. In total, 54 metabolites were quantified including amino acids, organic acids, sugars and a few miscellaneous compounds. **Figure 5** shows the metabolic changes in the *AtJUB1-OX* line in comparison to wild type, where the relative changes are depicted in blue (decreased) or red (increased) (complete data are given in **Supplementary Table S4**). Thirty-four metabolites out of 54 (∼63%) were significantly increased in the overexpression line compared to the wild type, with similar trends being observed at all three ripening stages. In general, the content of amino acids increased in the overexpression line compared to the wild type. Among these the levels of proline, β-alanine and phenylalanine showed considerable increases being a maximum of 12-, 14- and 7-fold higher in the *AtJUB1-OX* line compared to wild type. However, the greatest change was observed in the gamma-aminobutyric acid (GABA) content which was approximately 34-fold higher in ripe fruit of the *AtJUB1-OX* line compared to wild type fruit at the same stage (B+7). In addition, a marked increase was observed in major organic acids, particularly for glutamic acid (12-fold), aspartic acid (4.8-fold) and pyroglutamic acid (3.6-fold). Among the detected disaccharides, the levels of trehalose and maltose were mostly changed. By contrast, relatively few metabolites decreased in the three ripening stages in the overexpression line compared to wild type with only succinic acid, galactinol and tyramine displaying this behavior, although urea, glycerol, raffinose, glyceric acid and quinic acids were less abundant in the overexpression line compared to wild type in red ripe fruit (B+7).

## Discussion

In this study, we characterized the biological function exerted by the *Arabidopsis* NAC transcription factor AtJUB1 (JUNGBRUNNEN1) when expressed in the heterologous plant tomato (*S. lycopersicum* L. cv. Moneymaker). Similar to *Arabidopsis*, overexpression of *AtJUB1* in tomato resulted in reduced plant size, dark-green curled leaves, a delay in flowering, and reduced reproductive organ size compared to wild type (Shahnejat-Bushehri et al., 2016); in addition, fruit growth and ripening was delayed in *AtJUB1-OX* tomatoes. In *Arabidopsis*, *AtJUB1* regulates various growth-related genes by directly binding to their promoters: it represses expression of *GA3ox1* and *DWF4*, which encode key genes of GA and BR biosynthesis, respectively, and of *PIF4*, a growth promoting TF of the bHLH family. In contrast, AtJUB1 activates the expression of the DELLA genes *GAI* and *RGA*, which encode important repressors of growth. Collectively, the transcriptional control exerted by AtJUB1 leads to the characteristic GA- and BR-deficiency phenotypes observed for *AtJUB1* overexpressors, which is associated with a reduction of the levels of bioactive GAs and BRs and an accumulation of DELLA proteins (Shahnejat-Bushehri et al., 2016).

To gain insights into the mechanisms through which AtJUB1 affects growth in tomato, we analyzed genes orthologous to AtJUB1 targets in *Arabidopsis* (*GA3ox1, DWF4, GAI*, *RGL1*, and *PIF4*) in different tissues of *AtJUB1-OX* tomato plants and found that expression of all genes was altered, like in *Arabidopsis* itself.

In *Arabidopsis*, *GA3ox1* encodes a key enzyme involved in the biosynthesis of bioactive GAs, and it is expressed at relatively high levels at all stages of development (Mitchum et al., 2006); *ga3ox1* knockout mutants display a semi-dwarf phenotype due to reduced levels of bioactive GAs (Talon et al., 1990; Chiang et al., 1995). Here, we observed that expression of the *GA3ox1* tomato orthologs, *SlGA3ox1-1* and *SlGA3ox1-2*, was down-regulated in leaves as well as in three fruit developmental stages (MG, B and B+7) of *AtJUB1-OX* plants compared to wild type. The reduced expression of the rate-limiting GA biosynthetic enzymes may lead to lower levels of GA, thus contributing to GA-deficient phenotypes in *AtJUB1-OX* tomatoes similarly to *Arabidopsis* plants overexpressing *AtJUB1* (Shahnejat-Bushehri et al., 2016).

Furthermore, expression of *SlDWF4-1* and *SlDWF4-1*, orthologs of the *Arabidopsis DWF4* gene, was strongly reduced in leaves of *AtJUB1-OX* tomatoes, sharing this behavior with the repression of *DWF4* by AtJUB1 in *Arabidopsis* (Shahnejat-Bushehri et al., 2016). *DWF4* encodes a sterol C-22 α-hydroxylase that catalyzes a rate-limiting step in BR biosynthesis (Choe et al., 1998, 2001; Kim et al., 2006; Divi and Krishna, 2010); its reduced expression in tomato may contribute to the BR-deficiency growth characteristics observed.

Additionally, elevated expression was observed for the DELLA genes *SlGAI* and *SlGAI-like* in *AtJUB1-OX* fruits compared to wild type in the three developmental stages tested. While DELLA proteins are encoded by five genes in *Arabidopsis* (*GAI*, *RGA*, *RGL1*, *RGL2*, and *RGL3*) (Achard and Genschik, 2009), two DELLAs, namely SlGAI (identical to SlDELLA; Martí et al., 2007) and SlGAI-like, are encoded by the tomato genome (as deduced from the PLAZA 3.0 web database; Proost et al., 2015), and were analyzed here. GA promotes plant growth by removing DELLA proteins, whereas at low GA levels DELLAs accumulate and repress growth by directly inactivating several TFs including PIF4, a positive regulator of cell elongation (De Lucas et al., 2008; Feng et al., 2008; Arnaud et al., 2010; Leivar and Quail, 2011). In *Arabidopsis*, we found that AtJUB1 directly up-regulates *DELLA* genes thereby promoting their accumulation. Additionally, AtJUB1 represses the expression of *PIF4*, which further restricts cell elongation (Shahnejat-Bushehri et al., 2016). Similarly, reduced *SlPIF4* expression was evident in MG and B stage fruits of *AtJUB1-OX* tomato plants compared to wild type. Activation of *DELLA*s and repression of *SlPIF4* by AtJUB1 may contribute to the smaller fruit size of *AtJUB1-OX* plants.

In addition to the differential expression of GA-/BR-related genes in tomato upon *AtJUB1* overexpression, the presence of the AtJUB1 binding site in their 5′ upstream regulatory regions and the presence of an *AtJUB1* ortholog in the tomato genome and other plants (with in most case one predicted ortholog, see PLAZA 3.0^1^) strongly argue for a JUB1 regulatory network that has a similar architecture and function in *Arabidopsis* and tomato, and likely other plants. In support of our hypothesis, ChIP-qPCR revealed a significant enrichment of promoter regions of all tomato GA- and BR-related gene orthologous to the AtJUB1 targets in *Arabidopsis*, while no enrichment was detected for a promoter region lacking an AtJUB1 binding site.

Additionally, we were interested in understanding the molecular mechanisms associated with the delay of fruit ripening in *AtJUB1-OX* plants. Toward this end, we performed transcriptome analysis of *AtJUB1-OX* and wild type fruits at MG and B+7 stages. Genes differentially expressed in *AtJUB1-OX* fruits compared to wild type encode TFs, genes involved in ethylene synthesis or signaling, cell wall modification, flavonoid biosynthesis, peroxidases, and others. Of note, expression of several ethylene-related ripening genes such as *ACS* (ACC synthase) and *ACO* (ACC oxidase), *MADS-RIN*, and *SLERF.H15*, among others, was repressed in *AtJUB1-OX* tomatoes. Tomato is a climacteric fruit that requires an ethylene burst for normal fruit ripening (Alexander and Grierson, 2002). In fruits of several species, including, e.g., tomato and melon, inhibition of key enzymes of ethylene biosynthesis (ACO and ACS) by antisense RNA leads to strong delay in ripening, demonstrating the requirement of ethylene in the process (Oeller et al., 1991; Picton et al., 1993; Ayub et al., 1996). With respect to TFs, the MADS-box protein RIPENING INHIBITOR (RIN) has been particularly well-characterized. It exerts its function in fruit ripening by directly regulating the expression of genes involved in ethylene production, lycopene accumulation, chlorophyll degradation, among several other physiological processes (Fujisawa et al., 2013). Ripening is completely abolished in the *rin* mutant (Vrebalov et al., 2002).

Apart from *ACS* and *ACO*, a *PECTIN ESTERASE* gene (*Solyc06g051960*) is repressed in *AtJUB1-OX* fruits compared to wild type; similarly, several pectin esterase encoding genes are repressed in the *rin* mutant during fruit ripening (Kumar et al., 2016). The absence of the AtJUB1 binding site in the promoters of the ripening-related genes, however, indicates indirect regulation by AtJUB1; the details of the mechanisms that underlie this regulation are currently unknown.

With respect to metabolites, in general, similar significant changes were found in *AtJUB1 Arabidopsis* and tomato overexpressors: 67 and 63% of the metabolites detected were significantly different between wild type and overexpression lines, respectively. However, here (in tomato *AtJUB1* overexpressors) the relative changes were much higher than what we previously reported in *Arabidopsis AtJUB1* overexpressors (Wu et al., 2012). In tomato, 44 out of 53 metabolites were significantly increased and nine decreased at least in one developmental stage. On the other hand, in *Arabidopsis AtJUB1* overexpressors 21 metabolites were significantly increased and 17 decreased compared to wild type (**Table 1**). Comparing the metabolite changes indicates that 20% of the metabolites (nine increased and two decreased) overlapped and were significantly different in *AtJUB1* overexpression plants compared to wild type, in both, tomato and *Arabidopsis*. On the other hand, 17 metabolites (34%) found to be significantly different between overexpression and wild type plants showed different behaviors in tomato and *Arabidopsis*.

**Table 1:** Summary comparing the number of metabolites in *Arabidopsis* and tomato *AtJUB1* overexpression lines.

	Tomato	*Arabidopsis*
Number of metabolites	53	51
Significantly increased	44	21
Significantly decreased	9	17
Significant overlap – increased	9	
Significant overlap –decreased	2	
Significantly changed in both, but in different directions	17	

In tomato *AtJUB1* overexpressor plants proline showed one of the most drastic increases in overexpression lines at all developmental stages, which increased up to 12-fold higher than in the wild type. We made a similar observation in *Arabidopsis AtJUB1* overexpression plants where proline was increased up to twofold compared to wild type (Wu et al., 2012). Proline is known to be involved in the response to various environmental stresses. Among other metabolites that showed significant changes and similar trends to proline are trehalose, malic acid, and citric acid.

Another metabolite that had strongly increased levels in fruits of tomato *AtJUB1* overexpressors compared to wild type fruits is GABA, which was 4-, 8-, and 34-fold higher in the MG, B and B+7 stages, respectively (**Figure 5** and **Supplementary Table S4**). In wild type fruits GABA levels accumulate before the breaker stage, but shortly thereafter GABA is rapidly catabolized (Akihiro et al., 2008), most likely to support the high respiratory demand of later stages of fruit development. The fact that GABA content remains elevated in *AtJUB1* overexpressors is in accordance with the delayed fruit ripening observed in these plants. The biological function of GABA in tomato fruits is not well-known, but may include the regulation of pH to prevent cellular acidification during fruit development when organic acids are synthesized, or help to maintain glutamate import from source organs by converting it to GABA (through the action of glutamate decarboxylase) (reviewed in Takayama and Ezura, 2015). The high accumulation of glutamate (9- and 12-fold in B and B+7 fruits, respectively; **Supplementary Table S4**) in *AtJUB1* overexpressors compared to wild type supports this conclusion. Alternatively, GABA, which functions in defense against pests and pathogens might protect the immature seeds in developing fruits (Takayama and Ezura, 2015).

Also strongly elevated in *AtJUB1* overexpressor fruits throughout all developmental stages is β-alanine (10- to 14-fold higher than in wild type; **Figure 5** and **Supplementary Table S4**). Typically, β-alanine decreases during tomato fruit ripening (Carrari et al., 2006); the fact that its level remains high in *AtJUB1* overexpressors even at later stages of fruit development is also in accordance with the delayed ripening observed in these plants.

In addition, among the few metabolites showing a significant decrease in overexpression lines in tomato *AtJUB1* plants, raffinose (0.4-fold) and galactinol (0.2-fold), these metabolites decreased up to 0.6-fold and 0.2-fold in *Arabidopsis* overexpression plants compared to wild type, respectively. In contrast to *Arabidopsis AtJUB1* overexpression lines, some organic acids, in particularly glyceric acid and succinic acid, showed a significant decrease in the tomato *AtJUB1* plants (0.7- and 0.2-fold, respectively) compared to wild type. Succinic acid is a breakdown product of GABA and indeed is mobilized to augment flux through the TCA cycle (Studart-Guimarães et al., 2007); the low level of succinic acid in the presence of high GABA concentration (see above) indicates a reduced conversion of GABA to succinic acid in *AtJUB1* overexpressor fruits compared to wild type fruits.

An accumulation of GABA levels was previously observed in fruits of transgenic tomato plants in which expression of GABA transaminase (GABA-T) was suppressed by RNA interference. GABA-T converts GABA to succinic semialdehyde which is then further metabolized to succinic acid by succinic semialdehyde dehydrogenase. Interestingly, inhibition of *GABA-T* led to plant dwarfism, concomitant with the over-accumulation of GABA (Koike et al., 2013), similar to the metabolic and growth phenotypes we observed here for *AtJUB1* overexpressors. In addition, the excessive accumulation of GABA may negatively affect cell elongation (Renault et al., 2011), leading to the dwarfed phenotype of plants strongly overexpressing *JUB1*.

Taken together, we show here that the NAC TF JUNGBRUNNEN1, which we previously reported to control key elements of the GA/BR metabolism and signaling network in *Arabidopsis thaliana* (Shahnejat-Bushehri et al., 2016), exerts a similar control over orthologous genes in tomato. A key observation of our study is that in both plants, AtJUB1 negatively controls the expression of growth promoting genes (*GA3ox1*, *DWF4*, and *PIF4*), while it positively regulates the expression of growth repressing genes (*DELLAs*). This finding suggests that other, as yet unknown elements of the JUNGBRUNNEN1 growth control module are considerably conserved between species. Interestingly, *JUB1* is a single-copy gene in most sequenced plant genomes, including dicots and monocots, with only few exceptions; e.g., two *AtJUB1* orthologs are reported by PLAZA 3.0 for maize (*Zea mays*), poplar (*Populus trichocarpa*) and soybean (*Glycine max*), while three orthologs are reported for field mustard (*Brassica rapa*). The multiple regulatory roles that JUB1 exerts on central components of GA- and BR-related metabolism and signaling, which is key to the control of plant growth, suggests that JUB1 activity is finely tuned during growth and in different organs. Future research should focus on identifying further control elements of the JUB1 module in *Arabidopsis* and other species.

## Author Contributions

SB and BM-R conceived the idea for the study and supervised the work. SB and BM-R wrote the manuscript, with contributions from SS-B and AA. SS-B, AA, and NM performed the phenotype analysis; VT performed the confocal imaging; AA, NM, and VT performed the expression profiling; SS-B performed the ChIP-PCR experiments. SA and AF performed the metabolite profiling and analyzed the respective data.

## Conflict of Interest Statement

The authors declare that the research was conducted in the absence of any commercial or financial relationships that could be construed as a potential conflict of interest.

## Footnotes

1 http://bioinformatics.psb.ugent.be/plaza/

2 https://www.solgenomics.net/

3 https://www.ncbi.nlm.nih.gov/geo/

## Supplementary Material

FIGURE S1 ***AtJUB1* transcript in transgenic tomato plants. (A)** Presence of full-length *AtJUB1* transcript (arrow) in leaves of transgenic plants *OX1*, *OX2* and *OX3*, confirmed by PCR using primers specific for *AtJUB1*. **(B)** Presence of *AtJUB1* transcript in fruits and leaves of *AtJUB1-OX* (= OX2) tomato plants (arrow). Samples were taken from fruits of different developmental stages (MG, B, B+7). As expected, no *AtJUB1* transcript is detected in wild type tomato plants (WT).

TABLE S1 **List of primers**.

TABLE S2 **Expression of GA- and BR-associated genes**.

TABLE S3 **Gene expression in tomato fruits**.

TABLE S4 **Primary metabolites**.
