# Supplementary figures and images for: Arabidopsis NAC Transcription Factor JUNGBRUNNEN1 Exerts Conserved Control Over Gibberellin and Brassinosteroid Metabolism and Signaling Genes in Tomato
Source: Front Plant Sci. 2017 Mar 7;8:214. doi: 10.3389/fpls.2017.00214 (PMC5339236; doi:10.3389/fpls.2017.00214)

Supplementary Figure 1.

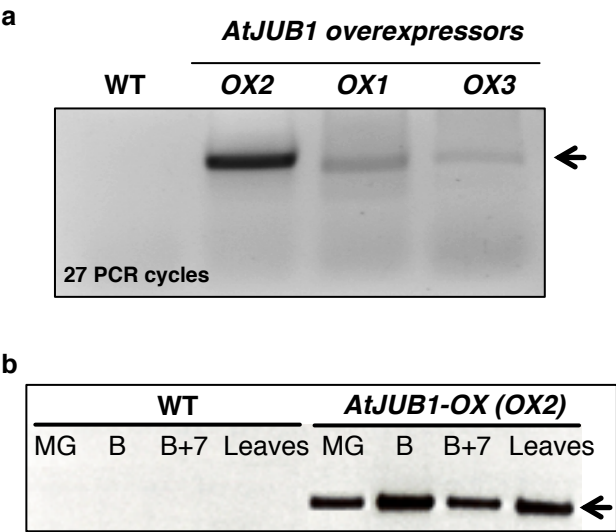

Supplement: FIGURE S1 — AtJUB1 transcript in transgenic tomato plants. (A) Presence of full-length AtJUB1 transcript (arrow) in leaves of transgenic plants OX1, OX2 and OX3, confirmed by PCR using primers specific for AtJUB1. (B) Presence of AtJUB1 transcript in fruits and leaves of AtJUB1-OX (= OX2) tomato plants (arrow). Samples were taken from fruits of different developmental stages (MG, B, B+7). As expected, no AtJUB1 transcript is detected in wild type tomato plants (WT). [file Image_1.PDF]
