# Supplementary material for: Arabidopsis NAC Transcription Factor JUNGBRUNNEN1 Exerts Conserved Control Over Gibberellin and Brassinosteroid Metabolism and Signaling Genes in Tomato
Source: Front Plant Sci. 2017 Mar 7;8:214. doi: 10.3389/fpls.2017.00214 (PMC5339236; doi:10.3389/fpls.2017.00214)
Supplement: TABLE S2 — Expression of GA- and BR-associated genes. [file Table_2.DOCX]

**Supplementary Table 2: Expression of GA- and BR-associated genes.**

**Data of Figure 3a.**

**qRT-PCR analysis of GA- and BR-associated genes regulated by AtJUB1 in tomato fruits and leaves.** Transcript ratio (log_2_ fold change) of orthologues of *Arabidopsis DWF4*, *GA3ox1*, *GAI,* *RGL1* and *PIF4* in line 24 at mature green (MG), breaker (B), breaker+7d (B+7) stages and leaves of mature plants compared to wild type (WT). All values are the mean of three independent experiments ± SD. Asterisks indicate significant differences between *AtJUB1* transgenic and WT plants. (**P* < 0.05, Students *t-*test).

| **AGI: Gene name** | MG | B | B+7 | Leaves |
| --- | --- | --- | --- | --- |
| SL02G085360 : DWF4-1 | -0.083±0.3 | -0.14±0.4 | -0.36±0.3 | -2.04±0.4* |
| 8SL04G080650 : DWF4-2 | -0.09±0.3 | -0.01±0.2 | -0.03±0.2 | -2.97±0.5* |
| SL06G066820 : GA3ox1-1 | -0.11±0.2 | -1.71±0.3* | -0.23±0.2 | -6.19±0.7* |
| SL03G119910 : GA3ox1-2 | -0.75±0.2* | -0.10±0.2 | -1.47±0.4* | -7.23±0.8* |
| SL11G011260 : GAI | 0.56±0.1* | 0.51±0.4 | 0.84±0.2* | 0.17±0.2 |
| SL01G009840 : RGL1-2 | 5.37±0.6* | 2.56±0.5* | 3.58±0.6* | 0.05±0.2 |
| SL07G043580 : PIF4 | -1.21±0.3* | -1.24±0.3* | -0.16±0.3 | -0.10±0.2 |
