# Supplementary material for: Arabidopsis NAC Transcription Factor JUNGBRUNNEN1 Exerts Conserved Control Over Gibberellin and Brassinosteroid Metabolism and Signaling Genes in Tomato
Source: Front Plant Sci. 2017 Mar 7;8:214. doi: 10.3389/fpls.2017.00214 (PMC5339236; doi:10.3389/fpls.2017.00214)
Supplement: TABLE S3 — Gene expression in tomato fruits. [file Table_3.DOCX]

**Supplementary Table 3: Gene expression in tomato fruits.**

**Data of Figure 4. qRT-PCR analysis of AtJUB1-regulated genes in tomato fruits.** Transcript ratio (log_2_ fold change) of genes differentially expressed in *AtJUB1-OX* fruits compared to wild type (WT). MG, mature green; B+7, breaker+7d. All values are the mean of three independent experiments ± SD. Asterisks indicate significant differences between *AtJUB1* transgenic and WT plants. (**P* < 0.05, Students *t-*test).

| **AGI: Gene name** | **MG** | **B+7** |
| --- | --- | --- |
| Solyc01g108240.2.1 : Redox responsive transcription factor 1 | -1.05±0.2* | -1.32±0.3* |
| Solyc03g026280.2.1 : CRT binding factor 2 | 1.82±0.4* | 0.20±0.3 |
| Solyc05g012020.2.1 : Ripening Inhibitor (RIN) MADS-box transcription factor | -2.38±0.4* | 0.43±0.4 |
| Solyc04g081000.2.1 : MADS box transcription factor | -2.96±0.5* | 1.81±0.4* |
| Solyc11g011050.1.1 : MYB transcription factor | 0.08±0.3 | -2.13±0.4* |
| Solyc06g050520.1.1 : Ethylene-responsive transcription factor 1 | -1.18±0.3* | 0.66±0.5 |
| Solyc05g056620.1.1 : Macrocalyx (mc) Myocyte-specific enhancer factor 2D | -1.32±0.3* | 0.91±0.2* |
| Solyc01g090460.2.1 : Homebox-leucine zipper protein (THOM1) | 2.11±0.4* | -0.20±0.3 |
| Solyc02g093050.2.1 : WRKY transcription factor 26 | 2.45±0.5* | 1.44±0.4* |
| Solyc03g116890.2.1 : WRKY transcription factor 2 | 4.07±0.6* | -1.41±0.4* |
| Solyc01g095630.2.1 : WRKY transcription factor 3 | 1.86±0.3* | -1.52±0.3* |
| Solyc04g051360.2.1 : Ethylene responsive transcription factor 2b | 1.40±0.4* | -2.91±0.6* |
| Solyc03g031450.2.1 : BHLH transcription factor-like protein | -2.77±0.7* | -0.30±0.3 |
| Solyc02g087840.2.1 : Homeobox-leucine zipper-like protein | 0.82±0.2* | -2.08±0.4* |
| Solyc10g080960.1.1 : Myb-like transcription factor | -0.87±0.2* | -0.79±0.2* |
| Solyc01g008180.2.1 : DNA-binding protein | 0.75±0.1* | -0.47±0.4 |
| Solyc06g069850.2.1 : MYB family transcription factor 306-like | 1.37±0.3* | -1.99±0.4* |
| Solyc07g055710.2.1 : Heat stress transcription factor A3 | 1.13±0.3* | 2.10±0.4* |
| Solyc08g021820.2.1 : Auxin responsive protein | 0.84±0.2* | 0.10±0.3 |
| Solyc02g037550.2.1 : Auxin efflux carrier family protein | -1.87±0.4* | -1.41±0.3* |
| Solyc02g082450.2.1 : Auxin efflux carrier family protein | -2.03±0.5* | -2.36±0.4* |
| Solyc11g069570.1.1 : Cytokinin riboside | -1.19±0.3* | -1.45±0.4* |
| Solyc06g082030.2.1 : Gibberellin 2-beta-dioxygenase 7 | -1.30±0.3* | 0.01±0.3 |
| Solyc10g084150.1.1 : Cytokinin riboside monophosphate phosphoribohydrolase | -3.97±0.6* | -1.69±0.4* |
| Solyc08g081550.2.1 : 1-aminocyclopropane-1-carboxylate synthase | 0.02±0.2 | -4.38±0.7* |
| Solyc02g036350.2.1 : 1-aminocyclopropane-1-carboxylate oxidase | -0.52±0.4 | -0.73±0.2* |
| Solyc07g026650.2.1 : 1-aminocyclopropane-1-carboxylate oxidase | 0.55±0.5 | -2.75±0.5* |
| Solyc11g072110.1.1 : 1-aminocyclopropane-1-carboxylate oxidase-like protein | -0.31±0.4 | -2.01±0.4* |
| Solyc12g056580.1.1 : Cellulose synthase | -1.11±0.3* | -1.98±0.4* |
| Solyc06g051960.2.1 : Pectinesterase family protein | -1.78±0.5* | -4.54±0.9* |
| Solyc11g005150.1.1 : Leucine rich repeat family protein/Extensin | 1.63±0.5* | -4.57±0.8* |
| Solyc06g051800.2.1 : Expansin 1 | -4.19±0.9* | 0.03±0.4 |
| Solyc06g076220.2.1 : Expansin 18 | 0.90±0.2* | -3.21±0.5* |
| Solyc02g088100.2.1 : Expansin | -1.47±0.4* | -3.02±0.6* |
| Solyc05g007830.2.1 : Expansin12 | -0.50±0.5 | -2.56±0.5* |
| Solyc01g006300.2.1 : Peroxidase | -1.63±0.4* | -4.83±0.9* |
| Solyc02g094180.2.1 : Peroxidase 1 | -0.13±0.3 | 1.37±0.3* |
| Solyc03g080150.2.1 : Peroxidase 1 | -2.28±0.4* | -3.46±0.7* |
| Solyc06g076630.2.1 : Peroxidase | -1.07±0.2* | -0.73±0.1* |
| Solyc01g105070.2.1 : Peroxidase | 1.72±0.3* | -0.75±0.1* |
| Solyc06g054260.1.1 : Photosystem I reaction center subunit II | -0.71±0.2* | 0.55±0.4 |
| Solyc09g014520.2.1 : Chlorophyll a-b binding protein 6A | -0.99±0.2* | 0.21±0.4 |
| Solyc10g006230.2.1 : Chlorophyll a-b binding protein 7 | -0.62±0.1* | -1.40±0.3* |
| Solyc12g006140.1.1 : Chlorophyll a-b binding protein 37 | -2.15±0.4* | -0.50±0.4 |
| Solyc12g011450.1.1 : Chlorophyll a-b binding protein 13 | -3.98±0.8* | -0.72±0.2* |
| Solyc05g052240.2.1 : Chalcone-flavonone isomerase | -0.89±0.2* | 1.51±0.4* |
| Solyc12g005350.1.1 : Dihydroflavonol-4-reductase | -0.24±0.3 | -0.78±0.2* |
| Solyc03g080180.2.1 : Flavone 3'-O-methyltransferase 1 | 0.18±0.4 | -1.07±0.2* |
| Solyc05g047530.2.1 : Cinnamade-4-hydroxylase | 2.09±0.4* | -2.55±0.6* |
| Solyc10g038080.1.1 : Shikimate dehydrogenase | -1.48±0.3* | 0.05±0.5 |
| Solyc08g062450.1.1 : Heat shock protein 17.6A | -3.27±0.6* | -0.61±0.5 |
| Solyc01g109130.2.1 : Proteasome assembly chaperone 2 | -0.89±0.2* | 0.59±0.4 |
| Solyc11g066100.1.1 : Heat shock protein 70 | -1.59±0.4* | 0.48±0.5 |
| Solyc01g067460.1.1 : Glutaredoxin family protein | 3.66±0.6* | 4.59±0.8* |
| Solyc09g011590.2.1 : Glutathione S-transferase-like protein | 1.94±0.5* | 1.91±0.6* |
| Solyc01g104400.2.1 : Blue copper protein | -0.45±0.5 | 2.58±0.6* |
| Solyc07g054210.2.1 : Protochlorophyllide reductase like protein | -0.91±0.2* | 0.57±0.6 |
| Solyc08g068730.1.1 : GCN5-related N-acetyltransferase | 0.84±0.1* | -1.17±0.2* |
| Solyc01g095140.2.1 : Late embryogenesis abundant protein | 2.53±0.7* | -2.86±0.6* |
| Solyc01g102330.2.1 : Acetyl xylan esterase A | 0.99±0.2* | -1.80±0.4* |
